# A natural language model to automate scoring of autobiographical memories

**DOI:** 10.3758/s13428-024-02385-5

**Published:** 2024-04-25

**Authors:** Meladel Mistica, Patrick Haylock, Aleksandra Michalewicz, Steph Raad, Emily Fitzgerald, Caitlin Hitchcock

**Affiliations:** 1 Melbourne Data Analytics Platform (MDAP), University of Melbourne, Melbourne Connect, Carlton, 3053 Victoria Australia; 2 Melbourne School of Psychological Sciences, University of Melbourne, Tin Alley, Parkville, 3010 Victoria Australia

**Keywords:** Autobiographical memory task, AMT, Large language models, Natural language processing

## Abstract

Biases in the retrieval of personal, autobiographical memories are a core feature of multiple mental health disorders, and are associated with poor clinical prognosis. However, current assessments of memory bias are either reliant on human scoring, restricting their administration in clinical settings, or when computerized, are only able to identify one memory type. Here, we developed a natural language model able to classify text-based memories as one of five different autobiographical memory types (specific, categoric, extended, semantic associate, omission), allowing easy assessment of a wider range of memory biases, including reduced memory specificity and impaired memory flexibility. Our model was trained on 17,632 text-based, human-scored memories obtained from individuals with and without experience of memory bias and mental health challenges, which was then tested on a dataset of 5880 memories. We used 20-fold cross-validation setup, and the model was fine-tuned over BERT. Relative to benchmarking and an existing support vector model, our model achieved high accuracy (95.7%) and precision (91.0%). We provide an open-source version of the model which is able to be used without further coding, by those with no coding experience, to facilitate the assessment of autobiographical memory bias in clinical settings, and aid implementation of memory-based interventions within treatment services.

## Introduction

Autobiographical memory of one’s personal past plays a key role in the formation of self-identity, and subsequently, mental health (Conway & Pleydell-Pearce, 2000; Dalgleish & Hitchcock, 2023; Williams et al., 2007). Models of autobiographical memory (Conway & Pleydell-Pearce, 2000) propose that autobiographical knowledge is stored in a fluid manner, such that prior experience can be recalled as generalized summaries which characterize categories of events (i.e., categoric memories) or extended periods of time (i.e., extended memories), or conversely, as specific, single-incident events which are isolated in space and time and contain a high level of detail (i.e., specific memories). The ability to retrieve specific memories has been implicated in the onset and maintenance of multiple mental health disorders (Barry et al., 2021; Hallford et al., 2021a), including the primary onset of symptoms (Askelund et al., 2019). Further, reduced ability to retrieve specific, single-incident memories appears to be a marker of higher chronicity of symptoms, as it is associated with increased frequency of depressive episodes and suicide attempts (Williams & Broadbent, 1986). As such, there is great potential for screening of autobiographical memory retrieval in both a preventative context, to indicate those who may be at risk of developing mental illness, and in treatment settings, to indicate those likely to experience chronicity and/or identify those likely to benefit from completing adjunctive memory-based interventions (Barry et al., 2019b; Dalgleish & Hitchcock, 2023).

A key barrier to widespread assessment of autobiographical memory retrieval is the scoring of the obtained memories, that is, the classification of responses as a given memory type. The gold standard for assessing memory retrieval is the Autobiographical Memory Task (AMT). This is traditionally scored by hand, by trained researchers. This reliance on human scoring limits how widely we can use the AMT, due to time constraints (each AMT takes approximately 5 min to score), along with the reductions in the accuracy of scoring caused by inter-rater variability and scorer fatigue. In response to this demand, Takano et al. (2019) developed an automated scoring system by training a support vector machine (SVM). However, this system simplified certain details of the original AMT, conflating general, summary memory types such that the prediction model only distinguished between the binary class of *specific* versus *non-specific*.

In the present study, a team of cognitive and clinical psychologists, data analysts, and computational linguists worked together to apply techniques employed in natural language processing (NLP) to develop a machine learning model able to classify text-based memory responses, advancing from binary classification to a multiclass model. This model expands the *non-specific* memory types so that there is a more fine-grained distinction of these memories. We provide an open-source tool which can be used in research to enable collection of very large datasets needed to answer important basic science questions regarding the processes through which autobiographical memory influences the development of mental illness. Further, the automated scoring model will facilitate quick and easy assessment of autobiographical memory within clinical services, thus taking important steps toward implementing a personalized approach (e.g., by identifying those individuals who might benefit from adjunctive memory interventions, in addition to usual care (Dalgleish & Hitchcock, 2023)) to treatment of mental ill health.

## Background

The AMT is a cued-recall task in which individuals verbally report or type out a memory that comes to mind in response to a cue word of neutral, positive or negative emotional valence (Williams & Broadbent, 1986). Original task instructions ask the individual to provide a specific, single incident memory in response to each cue word (Williams & Broadbent, 1986), however variations of the task have now been developed. This includes a Minimal Instructions version (Debeer et al., 2009), where participants are simply asked to retrieve ‘a memory’ (i.e., a specific event is not requested). This version has been demonstrated as more sensitive to reduced memory specificity in community-based samples. An Alternating Instructions version (Dritschel et al., 2014) requires the participant to alternate between retrieval of a specific memory in response to one cue, and a categoric memory in response to the next cue. That is, the task requests retrieval of both specific and categoric memories. The Alternating version seeks to index memory flexibility, that is, the ability to deliberately retrieve any memory type on demand, as prior research has suggested that poor mental health is characterized by reduced movement between specific and non-specific memory types (Hitchcock et al., 2018; Piltan et al., 2021). While early versions of the AMT involved a researcher delivering the task in person, with the participant verbally reporting their memories, later research has moved toward use of written AMT instructions and text-based reporting of memories to enable group-based and online delivery, and improve the ability to deliver the AMT, at-scale (though Wardell et al. (2021) have created a pipeline for transcription of verbally elicited responses into written form for scoring). For both verbal-report and text-based memories under each of these AMT versions, the classification of an individual’s reported memories has traditionally been reliant on human scoring of the response, using a coding manual. The coding manual (Williams & Broadbent, 1986) details five different categories of memory type; categoric, specific, extended (i.e., reference to events which took place for longer than 1 day, such as a holiday or semester), semantic associate (i.e., information related to the cue word which is not a memory), or omission (i.e., text indicating that no memory has been retrieved, e.g., ‘I don’t know’).

In the first effort to automate scoring of the AMT, Takano et al. (2019) developed a support vector model (SVM) involving linguistically motivated feature engineering, which is able to classify text-based memories as either specific or non-specific. The initial system was developed with Japanese memories, and has now been extended (Takano et al., 2018) for English-text memories written by children and English-, Dutch-, and Japanese-text memories written by adults (Takano et al., 2019). The model performs well. It reliably distinguishes between specific and non-specific responses, demonstrating the utility of a machine learning approach to scoring autobiographical memories. However, a key limitation of the SVM is the binary classification of a memory as specific or non-specific. That is, the current model is unable to identify categoric, extended, semantic associate, or omission memories. Moving from human scorers to the binary classification produced by existing SVM therefore reduces the richness of data. This is particularly important, as our research (Hitchcock et al., 2018; Piltan et al., 2021) has suggested that the ability to retrieve any memory type on demand may be a better characterization of the memory impairment experienced by those with poor mental health, than the ability to simply retrieve specific memories. Indeed, early research (Hitchcock et al., 2018) has suggested that interventions which target retrieval of a variety of memory types may potentially produce a larger effect on symptoms than the effect size commonly seen for interventions which train retrieval of specific memories alone (Barry et al., 2019b). Multiple research studies (Piltan et al., 2021; Mang et al., 2018) have therefore begun to use the Alternating Instructions adaption of the AMT to assess memory flexibility, that is, the ability to move between retrieval of specific and categoric memory types, as opposed to indexing retrieval of specific memories alone.

**Table 1:** Dataset characteristics: In Datasets C and D, education and employment were calculated from participant reports of whether they were best described as being currently in high school, tertiary education, taking time out for themselves, or employed

	Dataset A	Dataset B	Dataset C	Dataset D
	Takano et al. (2019)	Marsh et al. (2023)	(unpublished)	(unpublished)
Number of participants	1000	62	256	244
Number of responses	10,000	1488	6144	5880
Mean Age (SD)	34.70 (15.4)	39.08 (15.01)	20.31 (2.82)	20.30 (3.01)
Percentage female	60.30%	66.07%	74.31%	73.95%
Percentage Caucasian	78.80%	65.60%	52.50%	49.52%
Percentage completed or currently	65.40%	59.20%	50.00%^∗^	47.71%^∗∗^
completing tertiary education				
Percentage currently	64.70%	65.11%	14.29%	16.06%
employed fulltime				
Intra-class correlation	–	0.96	0.76-0.97	0.89
co-efficient for human raters				

In Dataset C, ^∗^ indicates that 30.95% of the participants were still in high school, while in Dataset D, ^∗∗^ indicates that 31.19% were in high school

### Applications of NLP to memory scoring

Methods applied within the field of NLP provide an unprecedented opportunity to improve consistency and speed of assessing clinically relevant cognitive processes. A recent systematic review of NLP methods applied to mental health assessment  (Zhang et al., 2022) indicated that most applications have focused on identifying or categorizing mental health symptoms. Analyzed texts have primarily been derived from social media (approximately 80%), with a smaller number of studies using text derived from clinical interviews (7%) or narrative writing (2%)  (Zhang et al., 2022). It therefore appears that there is considerable scope for expanding NLP applications to the large volume of text that is collected during clinical assessments and research. Doing so may not just refine ability to make diagnosis, as appears to be the aim of prior NLP applications, but also inform the selection of treatment components for each individual, thereby improving the personalization of mental health care. The automation of the AMT is an apt application for NLP and machine learning (ML) techniques.

In the recent decade, NLP has seen a seismic shift in the methodologies that are applied in solving text- and language-based problems. The shift derives from a few major contributions. First, there has been a drastic shift away from surface token representations as seen in more traditional machine learning models as implemented by Takano et al. (2017, 2018, 2019) to a more distributional representation (Pennington et al., 2014; Mikolov et al., 2013). This shift allowed the defining of tokens and sentences in terms of the context in which they appear, rendering identifying semantically related concepts a much easier task. In real terms, this means that if related but unexpected terminology is used, we would expect that their representations will be similar, if they are near synonyms, which gives rise to more robust systems. Second, advances in computer power allowed more intricate architectures to also be implemented, facilitating the application of deep neural networks in sequence-to-sequence problems (Sutskever et al., 2014) and paving the way for the development of large language models such as BERT (Devlin et al., 2019). BERT is a masked bidirectional language model that employs an attention model (Vaswani et al., 2017). Recently, van Genugten and Schacter (2022) implemented a fine-tuned system over DistilBert (Sanh et al., 2019), a more ‘compact’ version of BERT, to create an automated system that scores the internal, episodic details within autobiographical memories; a construct closely related to the ability to retrieve specific, single-incident memories (for discussion on the distinction between retrieval of discrete episodes, and the level of detail within memories, see Barry et al. (2021)). Our aim is to also leverage these more recent advances applied in the field of NLP and employ these large language models to create a full pipeline that can expedite the process of scoring the AMT.

**Fig. 1:**
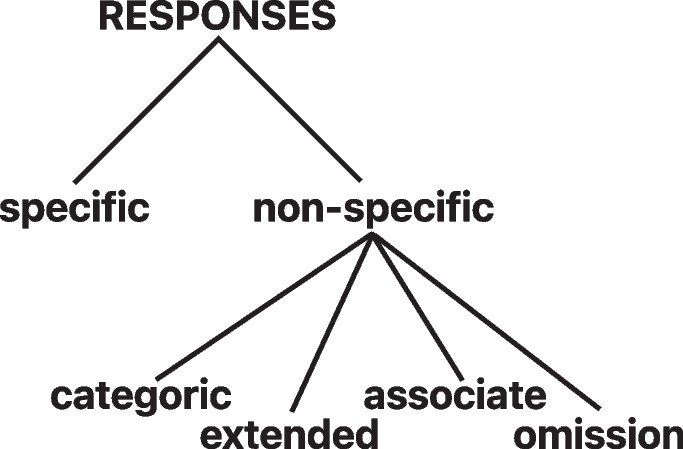
Breakdown of memory types used in the automatic classification of the AMT. Binarized classes are either *specific* or *non-specific*. Five-way classes expand the *non-specific* categories into *categoric*, *extended*, *associate* or *omission*

**Fig. 2:**
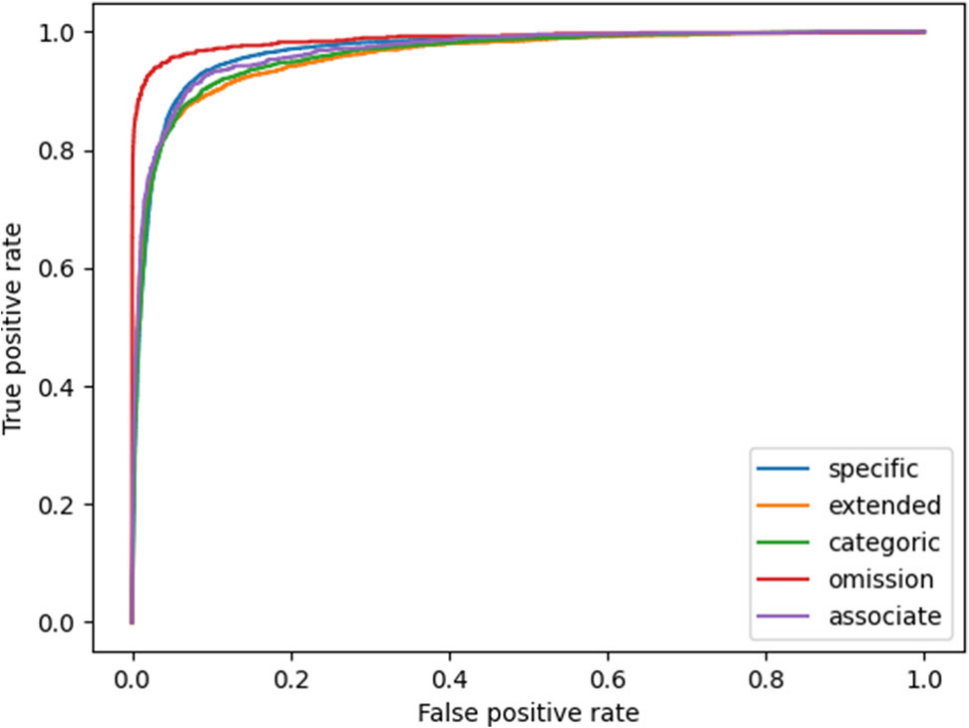
ROC curve using cross-validation for the ‘combined’ dataset in Table 3

## Method

In this section, we outline the datasets and methods we used in building the model. In the building of a model or achieving a resulting system, it requires two major elements: the data and the algorithm. We compare two types of algorithms. One is based on deep learning algorithms pretrained on large amounts of text data. We call these models *fine-tuned BERT models*, which we compare with our baseline models, which are traditional machine learning algorithms. Depending on the point of comparison of the resulting models or systems, the data or the algorithm may be highlighted or discussed, but each model required both components. All memories were obtained via a written version of the Autobiographical Memory Task (AMT) for which participants were provided with a positive, negative, or neutral cue word, and asked to type out a memory prompted by that word. The AMT is widely accepted as the gold-standard measure for autobiographical memory specificity, with factor analysis suggesting a one factor structure (Griffith et al., 2009). Responses to word cues were coded by human raters as one of five memory types: *specific* (i.e., an event that is located in time and place and lasted for less than 1 day), *categoric* (i.e., an event that happened on repeated occasion), *extended* (i.e., an event that occurred for longer than 24 h, such as a holiday), semantic *associate* (information that related to the cue but is not a memory), or *omission* (i.e., no memory provided).

### Data

We give a brief description of all the datasets used below. Dataset A (Takano et al., 2019) and B (Marsh et al., 2023) administered a minimal instructions version of the AMT, which did not ask participants to provide a certain memory type. Dataset C and Dataset D (unpublished) asked participants to provide a certain memory type in response to each cue.

For all datasets, the AMT was administered as part of a wider study which examined relationships between autobiographical memory retrieval and mental health. Sample statistics and properties (which may influence linguistic characteristics) for each dataset are presented in Table 1.

#### Dataset A: *takano*

The first dataset was 10,000 memories collected from 1000 USA-based participants by Takano et al. (2019) via Mechanical Turk.^1^ In response to ten cue words (five positive and five negative),^2^ participants were instructed to recall a personal event, and provide as many details as they can in relation to the event, but not to use an event from within the past week, or to repeat memories that had been mentioned for a prior cue word. This dataset was previously used by Takano et al. (2019) to train a SVM for scoring of the AMT.

#### Dataset B: *marsh*

For this dataset (Marsh et al., 2023),^3^ 62 individuals were recruited via the research volunteer panel of the MRC Cognition and Brain Sciences Unit, University of Cambridge, United Kingdom.^4^ All were experiencing a current major depressive episode. Data collection was completed online. In response to twelve cue words (six positive and six negative), participants were asked to provide an event from their personal past that the word reminded them of, but that it could not be from *today*. Participants completed the AMT twice, using different cue words, resulting in 24 memories per person for this dataset.

#### Dataset C: *amt-ai (tranche 1)*

For Dataset C, 256 individuals were recruited via online advertisements in Australia. This dataset is currently unpublished.^5^ The age range for this study was 16–25 years. Participants completed an online, Alternating Instructions of the AMT, which required specific memories in response to a block of six cues, categoric memories in response to a block of six cues, and for a block of 12 cues, to alternate between retrieval of specific and categoric memories. Positive, negative, and neutral cue words were randomized to the specific, categoric, and alternating blocks. Prior to the test trials, individuals were provided with a definition and example of a specific memory and a categoric memory. Participants were asked not to provide events from *today*.

#### Dataset D: *amt-ai-2 (tranche 2)*

This dataset is an extension of Dataset C: the same participants were invited back to complete another AMT using different cue words, 3 months later. As with Dataset C, these memories are currently unpublished.^6^ The same AMT task instructions were used. Of the 256 individuals in Dataset C, 244 participants provided AMT responses for this dataset. One participant provided two responses for this dataset, and all data from this individual were used.

### Experimental set-up

In this section, we outline how we prepared the datasets described in the previous section for the finetuning experiments detailed below. In addition, we detail our evaluation methods here so that the results reported in the next section can be easily interpreted.

#### Dataset preprocessing

While each dataset had their own classification scheme, they all followed the coding manual presented by Williams and Broadbent (1986) for the scoring of the AMT, and therefore could be directly mapped to one another. We normalized these datasets to align with the five-way schema followed by Dataset B (*marsh*). For the binarized version of the corpus (as used by Takano et al. (2019)) we combined the non-specific categories (*categoric*, *extended*, *associate* and *omission*) into one category, resulting in a *specific* versus *non-specific* distinction as illustrated in Fig. 1.

Data preprocessing also included removing any duplicates from the datasets (from when initial scorers had disagreed) and resolving these disagreements with a definitive outcome and manually correcting any missing data or errors. The small number of errors identified frequently came as a result of document formatting, such as misalignments that were corrected and other preprocessing issues were hand-checked and resolved.^7^

For the baseline systems (applying traditional machine learning), we used SpaCy^8^ with the appropriate large English language model^9^ to automatically perform tokenization, which we used to identify word tokens as features. Tokenization can identify sentence breaks from abbreviations, so that sentence-final words are not misconstrued as unique terms, and adding to data sparsity. These tokens were also case-folded^10^ for the same reason. For fine-tuned BERT models, we employed the accompanying tokenizer^11^ to preprocess all the text after duplicates and errors were manually corrected, as above.

#### Fine-tuning experiments

The models were fine-tuned over BERT (Devlin et al., 2019) using the bert-base-uncased model.^12^ The compute resources used in the training of the system had an Intel(R) Xeon(R) Silver 4214 CPU 2.20-GHz chip with an NVIDIA Tesla V100 SXM2 GPU with 32 GB GPU RAM.

For the data, we had a maximum sequence length of 512. Note that on the whole the AMT responses in this instance comprised of short answers of a couple of sentences. The batch size was set to 12 and the maximum number of epochs set to 20. For the initial training, we implemented warm-up steps, which allowed a lower rate of learning for the first 20$\%$ of the training steps. Warm-up steps help reduce the need for additional training epochs by slowly exposing the model to new data and possibly large variances in the data. While we had a maximum number of training epoch, we also implemented an early stopping criteria based on the validation loss calculated, using binary cross entropy, with a patience of 2.

For the experimental setup, we had a 20-fold cross-validation set up, which means at each fold 5% of the data was held out for testing while the other 95% was used for training and validation.

#### Baseline comparison

We compare the results from the fine-tuned model with a variety of baselines. The first is a weak majority class baseline. Although this is a weak baseline, it does allow us to determine how difficult the task is, and how the classes in the *gold standard dataset* are distributed. A *gold-standard dataset* in machine learning refers to the data used for evaluation. In this instance, our ‘gold-standard datasets’ are the human-scored memories described in the previous section under the heading ‘Data’. In addition, we compare this system to the SVM model developed by Takano et al. (2019) with the linguistically motivated features, and an SVM model without any feature engineering.^13^

**Table 2:** Results of the binary finetuning experiments

	DatasetA: *takano*			DatasetB: *marsh*			DatasetC: *amt-ai*			All: *combined*		
dataset size	10.0K			1.5K			6.1K			17.7K		
	p	r	f	p	r	f	p	r	f	p	r	f
Bert 0.45	.91	.91	.91	.92	.92	.92	.90	.90	.90	.89	.89	.89
Bert 0.50	.91	.91	.91	.92	.92	.92	.89	.89	.89	.89	.89	.89
Bert 0.55	.91	.91	.91	.92	.92	.92	.89	.89	.89	.89	.89	.89
Takano				.72	.71	.70	.72	.72	.71	–	–	–
SVM	.69	.71	.69	.61	.66	.63	.69	.68	.69	.78	.78	.78
Majority	.55	.55	.55	.50	.50	.50	.59	.59	.59	.50	.50	.50

There are no ‘All: *combined*’ scores for the Takano system because this dataset includes the same instances. Note that the grayed out figures showing results for the system developed over ‘Dataset A’ tested with the Takano data did not have training and testing mixed and were generously provided by Keisuke Takano via personal communication

**Table 3:** Results of the five-way multiclass finetuning experiments

	DatasetA: *takano*			DatasetB: *marsh*			DatasetC: *amt-ai*			All: *combined*		
dataset size	10.0K			1.5K			6.1K			17.7K		
	p	r	f	p	r	f	p	r	f	p	r	f
Bert 0.45	.91	.89	.90	.80	.83	.82	.94	.93	.93	.87	.87	.87
Bert 0.50	.92	.88	.89	.83	.81	.82	.94	.92	.93	.88	.85	.87
Bert 0.55	.92	.86	.88	.84	.79	.81	.95	.92	.93	**.91**	.86	**.89**
Takano	–	–	–	–	–	–	–	–	–	–	–	–
SVM	.78	.78	.78	.76	.76	.76	.78	.78	.78	.68	.68	.68
Majority	.55	.55	.55	.50	.50	.50	.41	.41	.41	.50	.50	.50

The bolded figures achieve the highest scores for precision (P) and overall f1-score (F) for BERT 0.55 with the Combined dataset. This model is made available

### Evaluation

We evaluate our systems both quantitatively and qualitatively so that future users of the model can gauge its strengths and limitations, and the degree to which they can rely on the output of the system. The qualitative analysis focuses more on the shortcomings of the model to better understand what types of inputs tend to lead to errors and why, and to guide practical use of model results.

#### Quantitative analysis

This section outlines the metrics we employ in our quantitative evaluation. Our first set of metrics is borrowed from information retrieval and the machine learning community. It is a set of three metrics called *precision*, *recall* and *f-score*, and the way in which they are calculated is shown in Eqs. 1, 2, and 3 below.

1 $$\begin{aligned} precision&=\frac{TP}{TP + FP} \end{aligned}$$

2 $$\begin{aligned} recall&=\frac{TP}{TP + FN} \end{aligned}$$

3 $$\begin{aligned} f1\text {-}score&=2 * \frac{precision * recall}{precision + recall} \end{aligned}$$

In Eq. 1 above, TP above stands for *true positive*. This represents the number of memories or responses that the model had correctly predicted as a certain category. In our case, it could represent the number of instances that the model had labeled ‘specific’ that was also labeled as specific in the gold-standard dataset. FP is *false positive*, which gives us the number of memories that the model had incorrectly marked as a particular category. The equation for *precision* has the sum of TP and FP as the denominator and TP as the numerator, and this metric helps us gauge the rate at which the system is correct when it commits to a prediction.

*Recall* in Eq. 2 differs slightly from *precision* because it measures ‘missed opportunity’. The denominator for recall has two elements as well: TP and FN. FN stands for *false negative*. If we take ‘specific’ again as an example, FN is the number of times the model should have marked a memory as ‘specific’ but did not. Therefore, *recall* measures the rate at which the model missed out on being able to identify and correctly mark a memory as specific.

The *f-score* defined above is a balanced harmonic mean of precision and recall. There are variations of the f-score which can favor either recall or precision, however we opt for the f1-score in our reporting which means we have an overall multiplier of 2 as shown in Eq. 3 above.

In addition to reporting on precision, recall and f-score, we gauge the performance of the model via the metrics Accuracy for testing the held-out dataset, and ROC (receiver operating characteristics) and AUROC (the area under ROC, when graphed) for showing the performance of our released model, which are in more standard usage in the field of psychology. Briefly, an ROC curve illustrates how the true positive rate (TPR) changes with respect to the false positive rate (FPR) for various threshold settings. TPR is calculated in the same way as recall in Eq. 2 above, and is also called *sensitivity*. FPR on the other hand is equivalent to $1 - specificity$, where *specificity* is calculated in a similar fashion to Eq. 1 but instead replacing TP with TN (true negative counts). AUROC (sometime referred to AUC), or calculating the area under the ROC curve, is a good indicator of a classifier’s performance, where 1.0 would be an impossibly perfect system, and 0.5 for a binary classifier would represent a random system. The closer to 1.0 AUROC is the better the model is at distinguishing classes.

#### Qualitative error analysis

For the qualitative analysis, we shift our focus to the errors that were made by the system so that we can better ascertain whether the model is fit for purpose. We identified all of the false-positive outcomes for each of the five categories (*specific*, *categoric*, *extended*, *omission*, and *associate*), and further grouped them into their correct classes, as deemed by the human scorers. In addition, we examined the output for when the model was not confident enough to make a prediction (i.e., it does not meet or exceed the specified threshold).

Two researchers manually reviewed these error files to determine whether the classification errors made by the model exhibited identifiable linguistic patterns. This qualitative procedure was performed iteratively over the course of three sessions to avoid mental fatigue.

During this process, however, the researchers identified inconsistencies in the originally scored memories. That is, in some cases where the model disagreed with the human scorers, our own scoring of the memory agreed with the model, rather than the original scorer. This may indicate that the model was identifying human-made scoring errors in the original dataset (e.g., made due to inattention or fatigue). Alternatively, this may reflect slight differences in the scoring manuals used between different research groups. For this reason, we performed an additional re-evaluation of a subset of our original collection of memories.

#### Further re-evaluation

For the instances where the model labeled a memory incorrectly, or where the human-scored memory and the model prediction disagreed, we had two researchers re-score a sample of these memories to ascertain the extent to which the model could be identifying human error in the initial dataset. Where there were model-to-human disagreements, we randomly obtained 250 of these instances for each of the classes. The rescoring was done in a two-stage process. In Stage I, the researchers were presented with a memory and two possible memory types to choose as the correct class. If the researcher deemed neither of the classes as correct for the given memory, they moved to Stage II, which allowed them to specify the correct memory type. The two possible memory types were taken as the prediction from the model and the original human-scored label. The researcher was not aware of which output was generated by the model or scored by the human.

#### Obtaining access to the model

We have made our final model freely available via GitHub, https://github.com/autobiographical-memory-task/amt-2023-08-01. We include step-by-step instructions on how to run the model. In doing so, we aim to make it simple for others to use the model to score their own data.

## Results

Table 2 shows the results for the binarized experiments where the four types of non-specific memories (*categoric*, *extended*, *associate* and *omission*) were conflated into one class, as shown in Fig. 1. Table 3 has the results for the multiclass experiments including all five classes: the four in parentheses above as well as *specific* ROC curve is displayed in Fig. 2.

### Baseline systems

For both sets of results, we have three kinds of baselines to compare our models to, which we call Takano, SVM and Majority. The latter is a simple majority class baseline. The other two baselines are SVM models. Takano is the R Model by Takano et al. (2019) that has linguistically motivated features that were designed especially for this binary task, and SVM does not involve any feature engineering; the text was simply tokenized and case-folded. The results for both the binary and multiclass systems convincingly outperform the baseline systems, in some cases achieving over 20 percentage points above the best-performing baseline. This is displayed in the results for the multiclass system for the *combined* dataset as shown in Table 3, which achieves an f-score of 0.89 when the threshold is 0.55 (BERT 0.55), while the comparison baseline system achieves an f-score of only 0.68 (SVM).

A noteworthy comparison is the results for the binary systems between Datasets B and C, *marsh* and *amt-ai*. We see that the Majority class baseline for *amt-ai* is 0.59 for the binary system and 0.41 for the multiclass system. This is because unlike the other datasets, *amt-ai* does not have the *specific* memory class that makes up a clear majority for the binary system – the baseline is made up of the heterogeneous class making up the *non-specific* memories. The other datasets have at least 50% of the one class, *specific*, that makes up the majority of the memories in the dataset. Therefore, while we expect that a system that is trained on more instances – or memories – to perform better than one that has fewer instances, we see that for the binary experiments, the *marsh* system outperforms *amt-ai*. This is primarily because the majority class does not form a homogeneous class with the same linguistic characteristics.

We observe in the multiclass results in Table 3 that the best-performing system is *amt-ai*. It outperforms the system trained on the *takano* dataset, even though the latter has 10K memories and the former has fewer with 6.1 K instances. This may largely be due to the make-up of the datasets. Dataset A, *takano*, had memories collected from diverse cohort whose average age was 34.7 years of age but with a standard deviation of 15.4 years, while the average age of *amt-ai* was almost 20 years, but with only a standard deviation of 0.67 years. We would expect that the linguistic style of the memories from *amt-ai* would be less diverse than that of *takano* for this reason.

The grayed out figures in the Takano baseline for the binarized task shows a precision, recall and f-score of 0.80, 0.82, and 0.81, respectively.^14^ It is interesting to note that the SVM model reported in Table 2 has not had any linguistically motivated feature engineering, unlike the Takano system based on an SVM model as reported by Takano et al. (2019). We can posit that the increase in performance between the systems SVM and Takano is due to the feature engineering designed by Takano et al. (2019).

### Fine-tuned BERT systems

We present three versions of our fine-tuned models based on a threshold of 0.45, 0.50, and 0.55. This means if the probability that the model predicts for a class exceeds this threshold then this prediction will hold. Otherwise, as a post-process, we disallow the model from committing to a prediction should the probability fall below. This thresholding does not result in a marked difference in the binary task, as shown by the consistent numbers in Table 2, as you read down each column representing the systems trained on each dataset. However, Table 3 illustrates that the thresholds increase precision for all four systems presented. For Datasets *takano* and *marsh*, this increase in precision as the threshold increases adversely affects the overall f-score due to the decrease in recall, but for Dataset *amt-ai* and the system that merges all three datasets (*takano*, *marsh*, and *amt-ai*) to form the *combined* system, we do not see a fall in the f-score. Furthermore, for the *combined* system, we see an increase in the overall performance for threshold 0.55, with precision, recall and f-score at 0.91, 0.86, and 0.89, respectively. In practical terms, this means that for every 100 predictions the system makes, it gets nine incorrect. However, the deterioration of the f-score (0.89) is largely due to poorer performance in the recall, our measure of ‘missed opportunity’.

We present the ROC curve for the best-performing multiclass system in Fig. 2. The closer the ROC curve hugs the upper left corner of the graph, the more proficient the model is in categorizing the data. To measure this, we can determine the AUROC (area under the roc curve), which reveals the portion of the graph that lies beneath the curve. This is depicted for each of the classes in the Fig. 2 where we see the class *omission* hugging the top left corner most closely with the further curve belonging to the *extended class*. Overall, the AUROC for the entire model for this five-way classification system has a value of 0.976 and an accuracy of 0.9572 (95.7%).

For the remainder of this section, we refer to the results from the system trained on the *combined* dataset with the threshold of 0.55^15^ in the ‘Error analysis’ and ‘Re-evaluation’ sections below.

**Table 4:** Breakdown of classes

	p	r	f	Data
Specific	.95	.92	.93	8802
Extended	.89	.77	.83	2852
Categoric	.86	.88	.87	4165
Omission	.99	.80	.88	687
Associate	.84	.66	.74	1113

### Error analysis

The aim of the qualitative error analysis was to identify what types of responses gave rise to errors in the model prediction. By doing so, this will allow users of the model to gauge the utility of the system. Table 4 provides a quantitative description of the *precision*, *recall*, and *f-score* metrics for each class. We observe that the model performs well in each of the *specific*, *extended*, *categoric*, and *omissions* classes, but the model exhibits poorer performance in the *associate* class. This quantitative error analysis coincides with this qualitative error analysis.

In the qualitative error analysis, we identified three main error types:

I Short responses;
II Present and future-oriented; and
III Ambiguous duration and frequencies

Error type I describes errors pertaining to short responses. We limited our analysis specifically to single-word responses, and these are typically best classified as semantic associates. The model was able to correctly classify many single-word responses as semantic associates, however it could also categorize these responses as categoric (“sleep”), omission (“Die”), none (“Drone”), extended (“retirement”) or specific (“funeral”). Human raters also exhibit similar errors in categorizing single-word responses, indicating that this may be an existing problem in scoring responses. Overall, single-word responses may benefit from re-scoring by carefully instructed human scorers. Further research on other short responses (e.g., two- and three-word responses) may be appropriate in future.

Error type II, *present and future-oriented* responses, is a result of the model mis-classifying responses that describe events that occurred ‘today’ or were anticipated to occur in the future. In these instances, the participant had not followed the experiment instructions to recall a memory from a period prior to ‘today’ (in Dataset B and C) or the previous week (in Dataset A). Under the scoring instructions used in each of the training datasets, these responses are treated as omissions (Takano et al., 2019). These responses can contain future and present markers (e.g., “today”, “will”), or hypothetical future scenarios (e.g., “If I don’t get this job that I am interviewing for”). The model can sometimes correctly identify these types of responses as *omissions* (e.g., “I will feel relieved after all the bills are paid.”) but the model also categorized these responses as *specific* (“today after dinner”), or *extended* (“Moving to New Zealand later in the year”), or *associate* (“Right now I’m bored”) or *categoric* (“When I have kids”).

Error type III describes errors pertaining to responses that have ambiguous duration and frequencies. The model could struggle to classify these responses in agreement with the human scorer. For example, the response “swimming with turtles” was interpreted by the model as a *categoric* memory, and as a *specific* memory by a human scorer. These types of responses are inherently challenging for both models and humans, as also observed in Takano et al. (2019). While a human scorer can use prior knowledge to reduce some ambiguity (e.g., for most people swimming with turtles is a rare occurrence), the use of world knowledge is often not decisive (e.g., ‘Going to a carnival" cannot be confidently classified as a specific or categoric memory).

While each error type is driven by unique features, we observed that each of these errors was also made by humans. As such, the model may partly reflect standard human scoring approaches. For example, type I errors (short responses) may be partly driven by some challenges humans scorers displayed in correctly categorizing single-word responses and semantic associates more generally, and type II errors (present and future-oriented responses) may be partly driven by similar difficulties humans exhibited in correctly scoring future and present responses as omissions, for example, “When I graduate college” was incorrectly scored by an annotator as a *specific* memory.

**Table 5:** Model comparison for binary systems

	SVM Model in R				Dataset A (0.55)				Dataset All (0.55)			
	p	r	f	Acc	p	r	f	Acc	p	r	f	Acc
B: *marsh*	.72	.71	.70	.71	.83	.82	.83	.83	−	−	−	−
C: *amt-ai*	.72	.72	.71	.72	.82	.83	.82	.83	−	−	−	−
D: *amt-ai-2*	.72	.72	.70	.72	.81	.81	.81	.81	.85	.85	.84	.85

Both the ‘SVM Model in R’ and ‘Dataset A (0.55)’ are developed from the *takano* (A) Dataset. ‘Dataset All (0.55)’ was developed from Datasets A, B, and C and tested on the held-out Dataset D

#### Re-evaluation

The process of the qualitative error analysis allowed us to recognize that in some instances the model had identified errors in the original manually scored corpus of memories and responses. In order to ascertain the extent to which the model identified human error, or whether these were isolated instances, we embarked on a process of re-evaluation as described in the Methods section (under ‘Further re-evaluation’). We had a primary annotator who re-evaluated 826 memories that disagreed with the human scores. A secondary annotator scored a large subset of these as a form of quality control.

Through this process, we found that the primary annotator agreed with the model’s prediction 64%; with the original human-scored annotation 31% of the time; and they rescored the response as a different category altogether 5% of the time. These proportions coincided well with the secondary annotator’s responses who agreed with the model 66% of the time, with the human annotation 31%; and disagreed with both 3% of the time.

The results of this re-evaluation demonstrate that, while the model generally agreed with the gold-standard human scored responses – as evidenced by the high-precision score – a majority of any disagreements once rescored by researchers actually found that the model picked up on human error. That is, the evaluation we have presented of the model may actually represent an underestimate of its true performance and may overcome errors that eventuate due to either annotation fatigue or shortcomings in the current human-driven scoring practices.

#### Error analysis summary

The error analysis identified three types of errors made by the model: error type I (short responses); error type II (present and future-oriented responses) and error type III (ambiguous durations and frequencies).

The error analysis also identified that a majority of disagreements between the model and human scorers were due to human-error. The model may therefore improve scoring rigor over existing human-driven practices.

#### Application to new data

In Table 5, we compare how our BERT-trained binary model fares against the system developed by Takano et al. (2019) labeled as ‘SVM Model in R’ on a completely new dataset. The system labeled ‘Dataset A (0.55)’ was trained on the same data as the ‘SVM Model in R’ but this model was fine-tuned over BERT. For the system labeled ‘Dataset All (0.55)’, there are no results for the first and second lines of the table to test both the *marsh* and *amt-ai* datasets because this system was trained on the memories from these corpora. The only completely held-out dataset was *amt-ai-2*, which we compare for all three systems in Table 5.

We observe that the held-out dataset fares the best for the ‘Dataset All (0.55)’ system, which was trained on three datasets. It performs over ten percentage points higher than the SVM model reported in the table for this dataset.

This shows that while the ‘SVM Model in R’ performs well, employing large language models that are fine-tuned outperforms traditional machine learning systems in this instance, with both ‘Dataset A’ (fine-tuned over the *takano* dataset) as well as our best-performing system fine-tuned on *takano*, *marsh* and *amt-ai*, validating that this methodology is apt for this application area.

## Discussion

Biased retrieval of autobiographical memories has been consistently demonstrated to predict depressive prognosis, and is increasingly recognized as feature of multiple other mental health disorders (Barry et al., 2021; Dalgleish & Hitchcock, 2023). Memory-based interventions show promise as a low-intensity treatment option (Barry et al., 2019a; Hitchcock et al., 2017), and can also be deployed as adjuncts to our current gold-standard treatments (Dalgleish & Hitchcock, 2023). Indeed, cognitive augmentations for psychological therapies are receiving increasing attention for boosting treatment effects (Nord et al., 2023). However, to effectively implement memory interventions into clinical services, we need fast, low-cost, and easy to implement tools for assessing memory bias, in order to effectively identify those who are likely to benefit from memory-based intervention. Further, if we are to better understand how memory bias influences treatment response, and the mechanisms through which memory bias impairs mental health, we need large-scale datasets. Here, we have developed a machine learning model able to identify multiple forms of memory bias, which can be used for these purposes. Critically, the model is open-source (i.e., does not require paid software), and does not require the user to have any coding experience, ensuring that it can be used by both clinicians and researchers alike, facilitating implementation and scalability within real-world settings.

Our developed model offers a number of advantages over existing scoring solutions. First, it is able to identify all five memory types in the original AMT scoring manual (Williams & Broadbent, 1986), facilitating the ability to identify not only reduced memory specificity, but also assess memory flexibility. The reliability and robustness of the model stems from the diversity of the data it is trained on with over 17,000 training instances from three diverse sources. This will enable further large-scale research examining the role of varied and non-specific memory types in the development of everyday cognitive skills (e.g., exploration of how categoric summaries of what usually works influence problem-solving skills). Second, our model was trained on text from both community-based and clinical samples collected from multiple countries, from participants aged 15 to 80 years, demonstrating that the model can be applied effectively to text composed by those with and without mental health disorders (Smirnova et al., 2018). Finally, we demonstrated an improvement in accuracy when using a large language model. Our preferred system (System All) outperformed previous machine learning approaches while also utilizing the more nuanced five-way schema as compared to a binary schema. Only 5.5% of responses fall below the 0.55 threshold and therefore require manual scoring, and researchers will be able to use the probabilities assigned by the model to facilitate the process. This model is therefore useful for the rapid assessment of autobiographical memory retrieval, in both a clinical and research setting.

The model also appeared to exhibit advantages when compared to human scorers. When the model and human scorers disagreed, a re-evaluation of these disagreements favored the model’s prediction approximately 65% of the time. This indicates that the model may be more robust to factors that may otherwise drive errors in human scoring. These factors include fatigue, which is not a concern for the model. Less obvious factors may include the additional information available to human scorers that the model does not have access to. Human scorers will often, when faced with ambiguity, refer to other information associated with the response including the associated cue words, the instructions provided (was the participant explicitly told to recall a specific memory?), patterns of responding in a single participant (does this participant generally provide specific memories?), and the scorer’s own experiences (are the described events common or unusual in their own life?). As such, many sources of information are available to the human scorer, which can reduce the focus on the content of the response provided. While under certain circumstances this additional information may confer an advantage (e.g., when resolving events with ambiguous durations), the focus on additional sources of information may contribute to variability in the human scoring processes. Our current model is trained on the scoring practices of a large number of different human scorers, across different countries, each with their own interpretive bias. The outcome of this training is that the model interprets responses in a manner that approximates the output of a large number and variety of human scorers. Thus, moving towards a machine scoring models likely minimizes multiple human biases in scoring.

In addition to variability between human scorers, there are likely consistent differences in scoring between research groups. Each research group appears to use slightly different judgments and thresholds when scoring ambiguous responses (e.g., whether ’benefit of the doubt’ is given, such that ambiguous responses are scored as correctly providing the requested memory type). Many of these small nuances in scoring arise from how new scorers are trained, and the errors that we have identified here (i.e., when the human scorer did not agree with the model) may not be considered as errors within some research groups. Datasets B, C, and D were all scored by our own research group, minimizing the impact of this issue on model training. While we did not observe any systematic differences in model accuracy between Dataset A (scored by another research group) and Datasets B, C, and D, small variation in scoring decisions between research groups will impact what is considered an error. When moving from human-led scoring to computer-led scoring, we encourage consideration of the likely systematic differences in the produced scores.

There are other limitations which will impact the generalizability of the model. We trained on data obtained from community samples or those with depression, and it will need to be determined whether the model applies as well to those experiencing other mental health disorders, which are associated with their own linguistic styles (e.g., psychosis). We also made use of memories which were reported in written form. Steady technological advances are facilitating automated text-based transcription of oral responses, allowing the future application of our model to transcribed, orally provided memories. However, it will need to be determined that model accuracy holds for orally reported memories, which likely vary in memory structure and length, from typed memories. Similarly, while data was obtained from numerous countries, all memories were in English. Reapplication of our code to new data will allow development of scoring models in languages other than English, and this is an exciting avenue for future research.

Future applications of this model may also wish to explore whether it can be used to accurately score future-oriented specific episodes. The ability to retrieve past episodes and project future events are closely related (Schacter & Addis, 2007), with indications that the ability to imagine specific future events is also associated with poor mental health (Hallford et al., 2018). Qualitative error analysis of our model suggested that the model may inconsistently identify future oriented episodes as omissions or specific. Further work to identify disagreements between human-scorer and machine in scoring of future episodes would produce a more detailed dataset that could be used for future training. Although we were focused on scoring memories for past events, and our model achieves high accuracy in doing so, additional further training may see that our model is able to be used in assessment of future thinking also.

Our model can now be used to evaluate research questions which necessitate the use of large datasets. Meta-analysis has indicated a significant prognostic effect of memory specificity on depressive symptoms across studies (Hallford et al., 2021b), however, there are indicators that the predictive effect may be stronger for certain participant groups (e.g., those with prior trauma exposure, a family history of depression) (Dalgleish & Hitchcock, 2023). Further understanding of moderators will help to identify who is most likely to benefit from memory-based interventions. Similarly, further understanding the mediating mechanisms underpinning the effect of memory specificity, or memory flexibility, on mental health outcomes is now needed. Evaluation of mediators and moderators will require datasets with hundreds of participants, assessed at multiple time points, which exceeds capabilities of human scorers. Our provided model therefore takes important steps toward enabling such necessary research.

The model also enables the secondary analysis of existing datasets. Current challenges in using existing data sets to complete new analyses often lie in the differences in how AMT responses are scored (e.g., in a binary or five-way schema) and reported (e.g., some researchers may combine categorical and extended memories into ’overgeneral memories’ Barry et al. (2021)). The current model enables existing datasets to be rapidly re-scored with a five-way schema and with high consistency, allowing for a more detailed secondary analysis of a broad set of autobiographical memory biases in existing datasets and for the pooling of these datasets to explore new research questions.

In terms of practical use, as indicated by our results, the model is likely to be of high utility due to its high accuracy. Lower, though still very good, accuracy was observed for semantic associates and thus this should be taken into account when interpreting results concerning semantic associates. Much of the research to date has focused on the ability to retrieve specific, categoric, and extended memories, and our results suggest that the user can be confident in model scoring of these memories. In providing a model that requires no additional coding, our goal has been to offer an easy to implement scoring system to assess autobiographical memory not only in future research, but also in clinical contexts. Increasing identification of those who are likely to benefit from autobiographical memory-based interventions is an important step toward clinical implementation of these basic science-driven interventions, and ultimately, personalization of mental health care.

## Open practice statement

All code, including instructions for use of the trained model, is available via GitHub, https://github.com/autobiographical-memory-task/amt-2023-08-01.

## Footnotes

1 Available via https://osf.io/ryshb/?view_only=1dc1501f2d1a4684822ea14ada15d995

2 A *positive* or *negative* word cue refers to either an adjective, noun or other open word class that is supposed to bring to mind a positive or negative experience, respectively. For example a positive cue could be *happy*, *joy*, etc., while an example of a negative cue could be *sad*, *loss*.

3 Available via https://osf.io/jqpek

4 https://www.mrc-cbu.cam.ac.uk

5 Dataset is pre-registered at https://osf.io/y79b3

6 The second tranche of this dataset is also pre-registered at https://osf.io/y79b3

7 These misalignments were only found in the exporting of our data, namely Dataset C and Dataset D. The results of these original assessments were exported to a spreadsheet format, however in the exporting of the files some of the cells in the spreadsheets misaligned.

8 https://spacy.io

9 The en_core_web_lg model is downloadable on the English models’ site:https://spacy.io/models/en

10 Performed the operation lower() ensuring all tokens as lower-cased

11 The BertTokenizer from huggingface: https://huggingface.co/docs/transformers/model_doc/bert#transformers.BertTokenizer

12 Available via https://huggingface.co

13 Employing the scikit-learn tools: https://scikit-learn.org/

14 These figures were provided by Keisuko Takano via private communication.

15 This model is available via https://github.com/autobiographical-memory-task/amt-2023-08-01
